# Obstetrics

**Published:** 1878-08

**Authors:** 


### Obstetrics.

Injection of Morphine in Incoercible Vomiting of Pregnancy.—Chabalier. (*Lyon Médical*, No. 22, 1878.)

The author describes an aggravated case of this affection, in which after the unsuccessful use of all the known means, he resolved to make use of hypodermic injections of morphine before the last resort, abortion. The patient was two and a half months pregnant. He injected the first time a little less than 0.016 Gm., which was followed by almost immediate relief; vomiting ceased and the patient was able to retain porridge, the first time in two months. But after the effect of the morphine had worn away, vomiting recommenced three or four hours after the injection. He continued its use, however, in doses of a little more than a third of a grain (2½ centigrams) morning and evening for two months, until the first movements of the child were felt. After each injection the patient was able to eat a tolerably good meal of soups, vegetables, meats, etc., and keep them sufficiently for maintaining nutrition. Vomiting came on several hours after the injections, but food was not vomited. On two occasions, wishing to suppress the morphine, and hoping that the moral effect would suffice, the author tried once pure water, and another time cherry laurel water; both times, although ignorant of the deception, the patient immediately vomited the food she had taken. Another time two drops of a one per cent. solution of atropine was used instead, which determined the most painful nervous excitement, which lasted all day, and aggravated the vomiting. At the end of four months and a half, on suspending the injections, the patient suffered all the effects of morphinism. For three days and nights she was in an indescribable state of agitation and excitement. There was absolute insomnia, and incessant involuntary movements of the limbs and body. At the end of this time all ceased, and the pregnancy pursued its normal course without accidents. Labor was natural, and the child presented no appearance whatever of having been affected by the action of the morphine.

The author has since tried this plan of treatment in two cases less severe, with the same success. He is convinced that if the method be courageously resorted to, not fearing the dose injected, the physician will not find himself disarmed before so grave an affection.

Eclampsia Relieved by Intra-venous Injection of Hydrate of Chloral. (*Ampiteatro Anatomico.*)—M. Bellmunt having failed to relieve a patient suffering from eclampsia by the internal administration of chloral hydrate, obtained speedy relief from an intra-venous injection of from 0.50 to 1 Gm. A dead fœtus was removed by forceps the same night but the mother made a good recovery.

